# Coffee Waste as a Green Precursor for Iron Nanoparticles: Toward Circular, Efficient and Eco-Friendly Dye Removal from Aqueous Systems

**DOI:** 10.3390/jox15050158

**Published:** 2025-10-02

**Authors:** Cristina Rodríguez-Rasero, Juan Manuel Garrido-Zoido, María del Mar García-Galán, Eduardo Manuel Cuerda-Correa, María Francisca Alexandre-Franco

**Affiliations:** 1 Departamento de Química Orgánica e Inorgánica, Facultad de Ciencias, Universidad de Extremadura, Avenida de Elvas s/n, 06006 Badajoz, Spain; cristinarr@unex.es (C.R.-R.); jmgarridoz@unex.es (J.M.G.-Z.); 2 Departamento de Dirección de Empresas y Sociología, Facultad de Ciencias Económicas y Empresariales, Universidad de Extremadura, Avenida de Elvas s/n, 06006 Badajoz, Spain; margalan@unex.es

**Keywords:** coffee waste, iron nanoparticles, dye removal, advanced oxidation processes, circular economy

## Abstract

In this study, the use of spent coffee waste as a green precursor of polyphenolic compounds, which are subsequently employed as reducing agents for the synthesis of zero-valent iron nanoparticles (nZVI) aimed at the efficient removal of dyes from aqueous systems, has been investigated. The nanoparticles, generated in situ in the presence of controlled amounts of hydrogen peroxide, were applied in the removal of organic dyes—including methylene blue, methyl orange, and orange G—through a heterogeneous Fenton-like catalytic process. The synthesized nZVI were thoroughly characterized by nitrogen adsorption at 77 K, scanning electron microscopy (SEM), transmission electron microscopy (TEM), Fourier-transform infrared spectroscopy (FT-IR), and powder X-ray diffraction (XRD). A statistical design of experiments and response surface methodology were employed to evaluate the effect of polyphenol, Fe(III), and H_2_O_2_ concentrations on dye removal efficiency. Results showed that under optimized conditions, a 100% removal efficiency could be achieved. This work highlights the potential of nZVI synthesized from agro-industrial waste through sustainable routes as an effective solution for water remediation, contributing to circular economy strategies and environmental protection.

## 1. Introduction

The circular economy is a transformative economic model designed to eliminate waste, circulate materials, and regenerate natural systems [1,2]. The traditional linear economy follows a “take–make–dispose” approach, where resources are extracted, used, and then discarded [1]. However, the circular economy employs different strategies focused on keeping products and materials in use, which involves reusing, repairing, remanufacturing, and recycling products and components for as long as possible to extract their maximum value [1,2].

Global waste generation was estimated at several billion tons per year and is projected to rise to 3 to 4 billion tons by 2050 if current trends continue. A significant portion of this waste, almost half, originates from the food and agricultural sectors [3]. In the global food industry, about 30% of production is lost or wasted, amounting to ~1.3 billion tons annually. Poor waste management pollutes soil, water, and air, threatening health and ecosystems. To address this, growing efforts focus on using food and agricultural residues as sustainable feedstocks for biorefinery products. Coffee waste is a particularly promising resource due to its low cost and high levels of lipids, carbohydrates, proteins, and bioactive compounds such as polyphenols and antioxidants [4,5]. Coffee, one of the most consumed non-alcoholic beverages, is produced from roasted *Coffea* seeds. With rising global demand, the industry generates large amounts of waste, especially spent coffee grounds, which are rich in bioactive compounds as noted above [6]. However, they can also pose environmental risks due to the presence of high levels of tannins and caffeine [7]. Notably, the polyphenolic compounds can interact with metal salt solutions and reduce them to form metal atoms. Furthermore, coffee beans contain two primary alkaloids, caffeine and trigonelline, along with other nitrogen-containing compounds like adenine, xanthine, hypoxanthine, and guanine [8]. These substances can facilitate the formation of nanostructures through oxidative coupling mechanisms. As a result, developing efficient methods for processing and utilizing food waste and by-products has become a critical global priority.

Studies have demonstrated the potential of synthesizing nanoparticles using plant extracts—such as green tea, rice straw, fruit peels, and coffee grounds—which contain key ingredients for nanoparticle synthesis, namely high concentrations of polyphenols [9] and iron sources that can catalyze dye degradation reactions, such as Fenton-like reactions [10]. These strategies are emerging as promising approaches to mitigate the environmental impact of highly polluting sectors like the textile industry, while also representing a transition toward cleaner and more responsible technologies.

The textile industry is a major source of wastewater containing synthetic dyes, heavy metals, and other chemicals. These pollutants degrade water quality, harm aquatic biodiversity, and can form toxic by-products with cumulative effects on organisms [11]. Water pollution—threatening a vital resource—remains a global challenge. Textile dyes are widely used in textiles, plastics, printing, rubber, leather, and paper, contributing significantly to wastewater contamination.

Nanotechnology, especially nanoparticle synthesis, offers promising solutions for dye removal. Nanoparticles (0–100 nm) with high surface area, stability, and biocompatibility are effective in wastewater treatment, bioremediation, biomedical research, filtration, cosmetics, and electronics.

Synthesis methods fall into two categories: top-down, which reduces bulk materials through ball milling, chemical etching, or sputtering, and bottom-up, which assembles nanoparticles from molecular precursors [12]. Bottom-up approaches, on the other hand, are typically based on chemical or biological methods, including sol–gel [13,14,15], coprecipitation [16,17,18], and hydrothermal synthesis [19,20], with chemical reduction being the most widely used method [21,22,23,24]. Zero-valent iron nanoparticles (nZVI) are of great interest due to their high surface-to-volume ratio, which places many atoms at the surface and makes them ideal for catalyzing chemical reactions [25]. Moreover, nanoscale zero-valent iron is considered one of the most suitable materials for water purification and the remediation of contaminated groundwater and soils [26]. The chemical reduction synthesis of these nanoparticles can be performed using sodium borohydride (NaBH_4_), a toxic and hazardous compound. However, plant extracts rich in reducing biomolecules can serve as a green alternative for nZVI synthesis, offering lower cost and eco-friendliness [27]. The biosynthesis of metallic nanoparticles has been investigated in recent years as an alternative to conventional synthesis methods.

This approach is sustainable, environmentally friendly, and more cost-effective than conventional chemical or physical methods. Biosynthesis often uses plant materials (e.g., leaves and bark) whose extracts contain antioxidants—mainly polyphenols—and smaller amounts of reducing sugars, nitrogenous bases, and amino acids. Nanoparticles form when these extracts contact metal solutions, reducing cations in place of NaBH_4_ used in chemical reduction. The morphology, size, aggregation, coating, and stability of the nanoparticles depend on both variable factors—such as biomolecule composition influenced by plant species and geography—and controllable parameters including solvent, temperature, pH, precursor salts, and stirring.

In this work, methylene blue, methyl orange, and orange G were selected as model pollutants because they represent different dye classes (cationic thiazine vs. anionic azo/sulfonated azo), vary in molecular structure, and are widely used in the textile industry. This diversity allows evaluation of the catalyst system under chemically distinct conditions. This work aims to remove the above-mentioned dyes through the green synthesis of zero-valent iron nanoparticles (nZVI) using coffee-waste polyphenols as reducing agents and supports, with iron salts as oxidants. The study examines how key parameters affect dye removal and the catalytic role of nZVI in Fenton-like reactions. Valorizing coffee waste as a feedstock promotes an innovative, sustainable process that recycles a common byproduct, reduces environmental impact, and supports clean technologies based on renewable, non-hazardous materials with inherent antioxidant properties. Beyond the statistical optimization of parameters, an important feature of this work is that nanoparticle synthesis and dye removal occur simultaneously rather than sequentially. This one-step approach exploits coffee-derived polyphenols as both reducing agents for nanoparticle formation and as facilitators of pollutant degradation, offering a distinctive and sustainable alternative to conventional two-stage processes.

## 2. Materials and Methods

### 2.1. Extraction and Determination of the Polyphenol Content of the Coffee Residue

Spent coffee grounds were collected from a local coffee shop in Badajoz, Spain, that consistently uses the same commercial brand of ground coffee for daily preparation. Although the supplier does not disclose the geographical origin of the beans, the use of a single brand ensured homogeneity of the raw material. Residues were collected immediately after use, dried at 40 °C to constant weight, homogenized, and stored in airtight containers until use.

Polyphenols were extracted by refluxing 5 g of dried coffee waste in 100 mL of deionized water at 80 °C for 20 min, following a reported protocol [28]. The mixture was cooled, filtered under vacuum, and the filtrate collected as the polyphenol-rich extract.

The method used for the qualitative analysis of natural extract has been described previously [29,30]. Samples were filtered through 0.45 μm nylon membrane filters. Polyphenols were separated by HPLC-DAD using a C18 column and standard gradient elution, with detection at 280 nm. Full operating conditions are provided in Supplementary Materials.

Polyphenols were identified by comparing retention times and spectra with authentic standards and quantified by external calibration (five-point curves from methanolic stock solutions). Measurements were performed in duplicate and expressed as mean ± SD (mg·g^−1^).

Because polyphenol content depends on coffee species, roast, and brew conditions, total polyphenol content was determined by the Folin–Ciocalteu method [31] and expressed as gallic acid equivalents (GAE). Their levels were adjusted experimentally to ensure comparability across assays. Also, to limit variability, all spent grounds were sourced from the same brand and preparation method. Experimental details are provided in Supplementary Materials (Section S1.2).

For future scale-up, higher phenolic recovery can be achieved with ethanol–water or acidified water, ultrasound/microwave assistance, and/or mild enzymatic pretreatments; however, a single aqueous extraction was used here to preserve simplicity and greenness.

### 2.2. Synthesis of Zero-Valent Iron Nanoparticles, n-ZVI, and Removal of Dyes. Influence of Variables

Nanoparticles were synthesized in situ during dye degradation tests. Dye solutions (50 ppm) of methyl orange, methylene blue, and orange G were prepared in a 75 mL total volume with added coffee extract to adjust polyphenol concentration. While stirring at 500 rpm, 25 mL of Fe(III) solution (Table 1) was added dropwise, producing a black, opaque suspension characteristic of nZVI formation. After 5 min of stirring, H_2_O_2_ was introduced and allowed to react for 15 min. The mixture was stirred for 5 min to ensure uniform dispersion and initial nanoparticle nucleation, after which H_2_O_2_ was added and allowed to react for 15 min. This reaction time was selected based on preliminary tests and literature, as dye removal by nZVI/Fenton-like systems is rapid and typically plateaus after 15–20 min.

The suspension was then centrifuged at 5000 rpm for 10 min, which yielded a clear supernatant and a black pellet of nanoparticles. The supernatant was analyzed spectrophotometrically to determine dye concentration and removal efficiency. Completeness of separation was confirmed visually and by checking that blank supernatants showed no measurable scattering in the 600–800 nm range.

### 2.3. Characterization of the n-ZVI

Samples were characterized in terms of their porous texture and morphology, as well as their chemical composition and crystal structure.

#### 2.3.1. Texture and Morphology

Textural characterization was carried out by nitrogen adsorption at 77 K, using a Quantachrome Autosorb-1 semi-automatic apparatus to obtain the N_2_ adsorption isotherms. To perform this analysis, about 0.15 g of carbon sample was deposited in a glass sample cell and was degassed at 250 °C for 12 h under vacuum conditions (pressure < 10^−3^ Torr).

The micropore volume (V_mi_) was calculated from the measured adsorption isotherms by taking the volume of nitrogen adsorbed at a relative pressure (p/p^0^) of 0.10. Furthermore, the mesopore volume (V_me_) was calculated using the N_2_ adsorption isotherm, with the formula V_me_ = V_0.95_ − V_mi_. Here, V_0.95_ denotes the volume of nitrogen adsorbed at a relative pressure of 0.95. The liquid volumes of V_mi_ and V_me_ were expressed using a conversion factor of 1.543 × 10^−3^.

The BET method was used to estimate the specific surface area (S_BET_), while the Dubinin-Radushkevich equation was used to obtain the micropore volume (W_0_).

The samples’ morphology was analyzed using Scanning Electron Microscopy (SEM) and Transmission Electron Microscopy (TEM). Images of the samples were taken using a field-emission scanning electron microscope (FESEM) JSM-7800F Prime model, made by JEOL Ltd. in Tokyo, Japan, coupled to an EDX analyser for the mapping of elements.

High-resolution images were collected using a transmission electron microscope (TEM, Jeol JEM 1400, Tokyo, Japan). The samples were dispersed in water using ultrasound before being deposited on a holey carbon grid.

#### 2.3.2. Chemical Characterization and Crystal Structure of nZVI

The surface chemistry of the samples was analyzed by FT-IR spectroscopy using a PerkinElmer 1720 spectrophotometer equipped with an ATR (attenuated total reflectance) accessory (PerkinElmer Scientific Spain S.L., Tres Cantos, Madrid, Spain). Spectra were collected from 650 to 4000 cm^−1^ at 4 cm^−1^ resolution, with fifty scans per spectrum.

X-Ray Diffraction (XRD) was used to investigate the crystal structure of the samples. Diffraction patterns were acquired using a Bruker D8 ADVANCE diffractometer equipped with a Vantec PSD detector (Bruker España S.A.U., Paterna, Valencia, Spain). The XRD analysis was carried out within the 2θ range of 15° to 80°, with a step size of 0.02°. This analysis used Cu Kα radiation (λ = 1.54184 Å).

### 2.4. Experimental Design

A factorial, composite, central, orthogonal, and rotatable (FCCOR) experimental design was used to investigate the impact of three operational variables on the efficiency of dye removal. The variables in question were the concentrations of polyphenols, Fe (III), and H_2_O_2_. The design comprised 8 factorial experiments, 6 axial experiments, and 9 replicates of the central experiments, giving a total of 23 experiments in the experimental matrix. Table 1 shows the experimental matrix, together with the removal efficiencies achieved for each of the three dyes in every experiment.

To facilitate interpretation of Table 1, Table S1 (Supplementary Material) reports the coded and corresponding actual levels of the operating variables.

### 2.5. Computational Methods

Optimizations of dye molecules in their ionic form have been performed using DFT (Density Functional Theory) calculations with Gaussian16 [32], applying the M06-2X method [33] and the 6-311++G(3df,3pd) basis set. Water has been considered as the solvent through the SMD (Solvation Model based on electronic Density) implicit solvation method [34]. Based on the molecular optimization data obtained from DFT (Tables S2–S5), we calculated the Fukui functions [35,36] using the Multiwfn 3.8 tool [37,38] and visualized the isosurfaces with the VMD tool [39].

## 3. Results and Discussion

### 3.1. Determination and Quantification of Polyphenols in Coffee Waste Extracts

**Figure 1:**
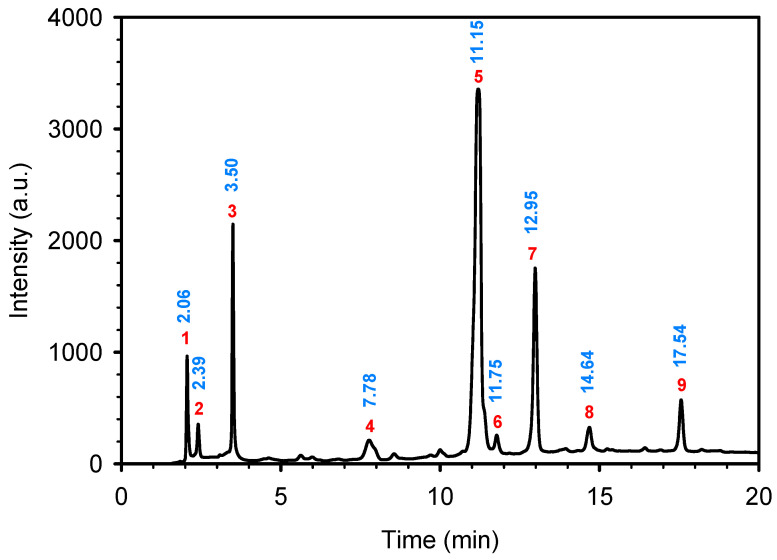
HPLC-DAD profile of coffee waste extract. Peaks numbered sequentially for correspondence with the numbering in Table 2.

Analyzing the polyphenol content of the coffee waste extract is crucial for green nZVI synthesis, as it establishes the effectiveness of the extract as a reducing agent [40]. The HPLC chromatogram of coffee waste extract (Figure 1) revealed nine major phenolic compounds eluting between 2 and 18 min, with no significant peaks detected afterward. Peak identities were confirmed with standards (Section 2.1). The profile was dominated by chlorogenic/caffeic species, followed by hydroxybenzoic and hydroxycinnamic acids, consistent with reversed-phase C18 retention.

The identification and assignment of the peaks in the chromatogram are included in Table 2.

Variations in coffee origin/roast can alter the phenolic profile; normalization by GAE was therefore used to compare experiments independently of feedstock nuances. The total polyphenol content of the coffee waste extract was 698 ± 18 mg GAE·L^−1^, corresponding to 13,960 ± 360 mg GAE·kg^−1^ dry waste. These values agree with recent reports [41]. Although variable in composition, residual polyphenols—though modest in economic value—play a key role in green synthesis and environmental sustainability.

### 3.2. Preparation and Characterization of the Zero-Valent Iron Nanoparticles, n-ZVI

#### 3.2.1. Porosity and Morphology

An adequate analysis of texture and morphology is essential to understanding the physical characteristics of the synthesized zero-valent iron nanoparticles (nZVI) [42]. This section examines the surface texture, pore structure and morphology of nZVI.

**Figure 2:**
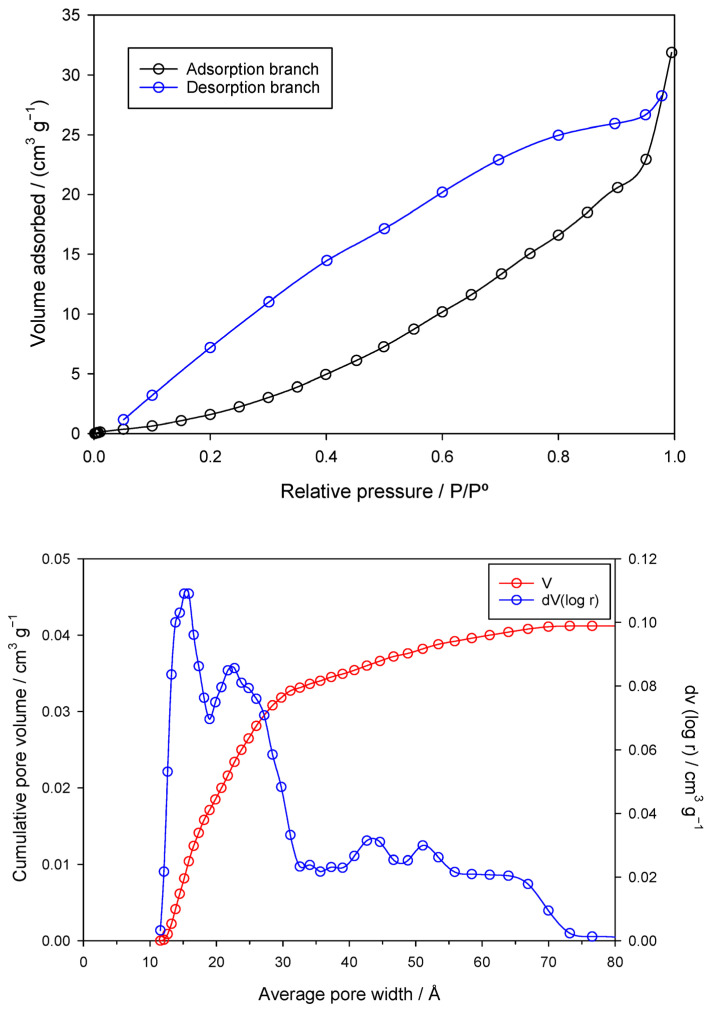
Nitrogen adsorption–desorption isotherm (**top**) and DFT pore size distribution (**bottom**) of the nZVI sample.

Figure 2 (top) shows the N_2_ adsorption isotherm of the nZVI sample at −196 °C.

The isotherm is convex to the relative pressure axis with no evident inflection point, characteristic of a Type III isotherm and weak adsorbent–adsorbate interactions, i.e., low affinity for nitrogen. Adsorption occurs mainly through cooperative adsorbate–adsorbate interactions, becoming significant only at higher pressures, while uptake at low pressures is minimal. Such behavior is typical of nonporous or weakly porous materials, or of surfaces that are hydrophobic or energetically heterogeneous.

The hysteresis loop extends from P/P_0_ ≈ 0.05 to 0.97, unusually broad for a Type III isotherm, and indicates a wide pore size distribution, dominated by mesopores. Its H3 shape suggests slit-shaped pores between plate-like particles and aggregated structures with poorly defined networks. Persistence of hysteresis at low pressures (P/P_0_ ≈ 0.05) further implies the presence of small mesopores.

The material is predominantly mesoporous, with V_me_ = 0.034 cm^3^/g compared to V_mi_ = 0.001 cm^3^/g, mesopores accounting for >83% of VT = 0.041 cm^3^/g. The DR method gave a higher micropore volume (W_0_ = 0.027 cm^3^/g) than the single-point estimate at P/P_0_ = 0.10, reflecting gradual micropore filling over a broader range. The low specific surface area (9 m^2^/g) supports limited microporosity and surface roughness. The average pore width of 16 Å places pores at the lower mesopore range, close to micropores, consistent with the broad hysteresis loop observed (Figure 2).

**Figure 3:**
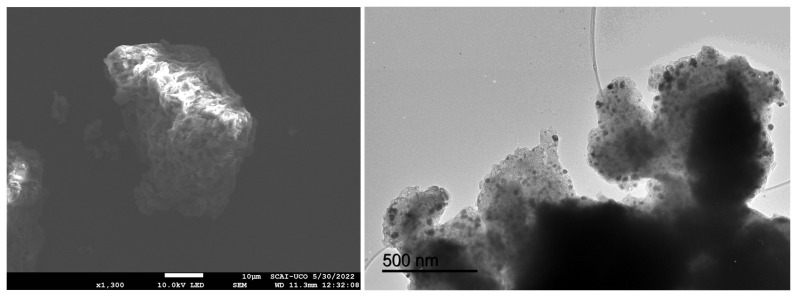
SEM (**left**) and TEM (**right**) images of the nZVI samples.

Figure 3 depicts the SEM (left) and TEM (right) images of the nZVI samples prepared from the Fe(III) precursor in the presence of the polyphenols contained in the coffee waste extract.

SEM images show irregular, cauliflower-like agglomerates with corrugated surfaces, indicating that nZVI formed secondary structures rather than isolated particles—consistent with magnetic dipole–dipole interactions and partial oxidation. TEM reveals these agglomerates are composed of near-spherical primary nanoparticles (tens of nm) arranged in grape-like clusters. The dense particle interiors with faint rims suggest a thin Fe^0^/Fe-oxide core–shell formed during work-up. Polyphenols from the coffee extract acted as reductants and weak capping/bridging agents: they restricted growth to nanoscale dimensions but did not fully prevent aggregation. This architecture enhances surface area and reactivity but may reduce colloidal stability and mobility.

#### 3.2.2. Chemical Characterization and Crystal Structure

An adequate characterization of the chemistry and crystal structure of the sample helps to demonstrate the successful synthesis of nZVI with desirable properties for environmental applications. With such an aim, the nZVI samples were first analyzed by EDX for the mapping of elements. As usual, EDX mapping was performed simultaneously with the acquisition of the SEM images, but it is discussed here as this analysis refers to the chemical composition of the external surface of the nZVI samples.

**Figure 4:**
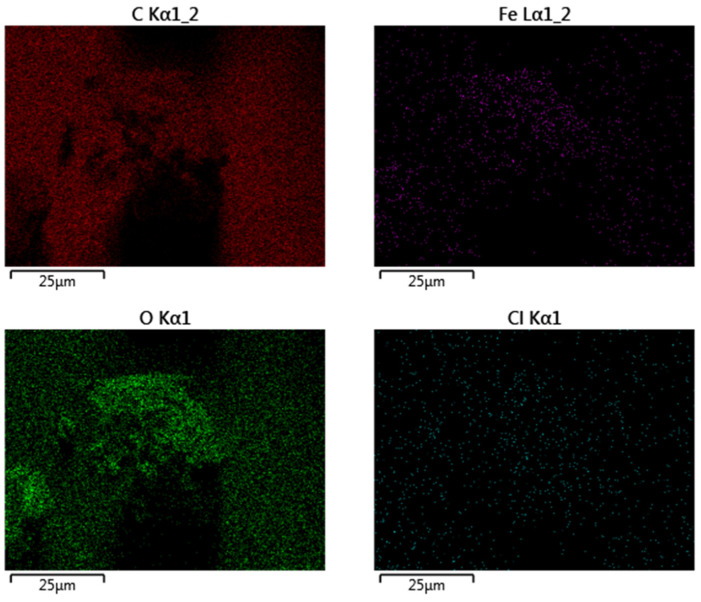
EDX mapping of the nZVI samples.

Figure 4 presents EDX mapping of nZVI, consistent with SEM images. Fe is concentrated in aggregates, with O co-localized and enriched at the periphery, indicating an Fe^0^/FeOx core–shell formed during work-up. C appears as a diffuse background with local enrichment around aggregates, suggesting a polyphenol-derived corona acting as a weak capping layer. Low, heterogeneous Cl signals reflect residual Fe(III) precursor, largely removed by washing. Overall, the imaging supports an Fe^0^/FeOx core–shell partially coated by polyphenolic species: polyphenols restricted growth to nanoscale dimensions but did not prevent aggregation. This structure favors interfacial reactivity but limits colloidal stability.

**Figure 5:**
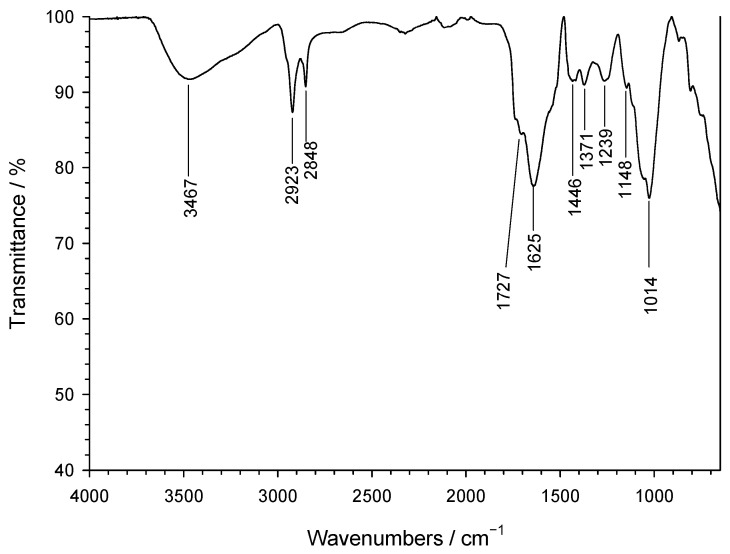
FTIR spectrum of the nZVI samples.

On the other hand, FT-IR spectroscopy [43], as illustrated in Figure 5, reveals a wide variety of spectral features.

The band at 1014 cm^−1^ and the shoulder at 1148 cm^−1^ correspond to in-plane C–H vibrations of aromatic rings, consistent with polyphenols in the coffee extract. The 1239 cm^−1^ band is attributed to arC–OH stretching, while the 1446 cm^−1^ band reflects –OH deformation. The band at 1625 cm^−1^ and the shoulder at 1727 cm^−1^ are assigned to C=C stretching in aromatic rings and C=O in phenolic esters, respectively. Bands at 2848 and 2923 cm^−1^ correspond to –CH_2_– stretching, with the 2848 cm^−1^ band also matching –O–CH_2_– in non-cyclic ethers. The broad 3467 cm^−1^ band indicates hydroxyl groups from polyphenols or –OH in FeOOH.

**Figure 6:**
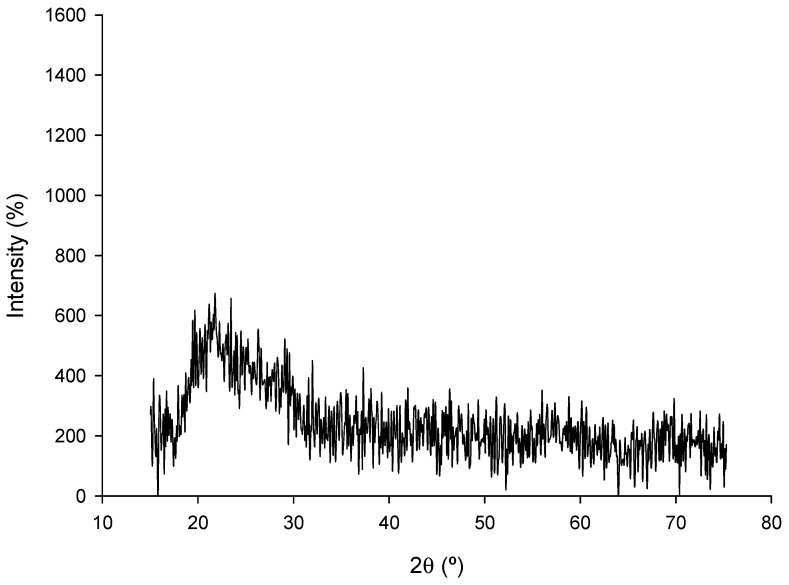
X-Ray diffractogram of the nZVI samples.

Regarding the crystal structure of the nZVI samples, Figure 6 shows the XRD diffractogram of the nanoparticles.

The XRD pattern of nZVI shows no sharp Bragg reflections but a broad halo at 22–28° 2θ. The α-Fe (110) peak near 44.7° and principal oxide peaks at 30.1°, 35.5°, and 62.6° are absent, indicating largely amorphous or poorly crystalline material, or nanometric domains with peaks too broadened to detect. The diffuse hump is consistent with an organic/carbonaceous matrix and a disordered oxide passivation layer. The noisy baseline likely arises from iron fluorescence under Cu Kα radiation. Combined with SEM, TEM, and EDX, the data support an Fe^0^–FeOx core–shell in which zero-valent iron is highly dispersed and below XRD detection.

The synthesis is enabled by polyphenols in coffee extracts, which contain aromatic rings with hydroxyl groups providing strong reducing power and chelating ability. Thus, nanoparticle formation proceeds mainly via ferric ion reduction by polyphenols, with minor contributions from other reductants. Polyphenolic –OH groups also form Fe–polyphenol complexes (Fe–P NPs), as reported in related plant-extract syntheses [44], yielding amorphous nanomaterials with sizes of a few tens of nm.

### 3.3. Removal of Organic Dyes from Water. Influence of Variables

To evaluate the effects of three factors—initial concentrations of polyphenols (as gallic acid equivalents, GAE), Fe(III), and hydrogen peroxide (H_2_O_2_)—on the removal efficiency of the three dyes, the experimental design described in Section 2.5 was applied. The resulting data were analyzed in two complementary parts: first by numerical analysis and then by graphical analysis. The results of such analyses are detailed below and will be discussed in depth. Nonetheless, it is worth noting that the present work was carried out using model dye solutions, which allowed systematic statistical evaluation of the main parameters. However, real textile wastewater contains salts, surfactants, and organic matter that may scavenge radicals or affect nanoparticle stability, potentially reducing efficiency. Future work should therefore assess the performance of coffee-waste-derived nZVI in real or simulated wastewater matrices to validate the applicability of the process under practical conditions.

#### 3.3.1. Numerical Analysis

The numerical analysis is structured into three parts: analysis of variance (ANOVA), derivation of a regression equation with corresponding evaluation of its correlation coefficients, and determination of the experimental conditions corresponding to the optimum of the response variable.

Table 3 lists the *p*-values obtained when performing for each of the operational variables, their quadratic terms and interactions.

Table 3 shows that significant main effects and interactions were identified for each dye, with model fit indicators confirming strong explanatory power. For methylene blue, Fe(III) and H_2_O_2_ were significant (*p* < 0.001), while polyphenols were not (*p* = 0.0576); AB and BC interactions were significant, AC was not. For orange G, polyphenols and H_2_O_2_ were significant (*p* ≤ 0.001), Fe(III) was not (*p* = 0.0894), with AB and AC interactions significant. For methyl orange, Fe(III) and H_2_O_2_ were significant, polyphenols were not, and a strong BC interaction was detected. Model fits were high (R^2^ = 99.67% MB, 93.90% OG, 96.64% MO) with adjusted R^2^ values close to raw R^2^. No residual autocorrelation was detected, and mean absolute errors were low. Overall, the quadratic models are adequate for effect interpretation and prediction.

The regression equations are shown below for each of the dyes under study.

(1) $$\begin{array}{l} Removal\;efficiency\;MB\;\left(\%\right)\;\\ \;=90.8016-0.584658\cdot \mathrm{Polyphenols}+5.84132\cdot \mathrm{Fe}\left(\mathrm{III}\right)+11.3526\cdot H_{2}O_{2}+2.46541\\ \;\cdot \;{\mathrm{Polyphenols}}^{2}-1.7336\cdot \mathrm{Polyphenols}\cdot \mathrm{Fe}\left(\mathrm{III}\right)+0.59375\cdot \mathrm{Polyphenols}\cdot H_{2}O_{2}-2.52489\\ \;\cdot \mathrm{Fe}{\left(\mathrm{III}\right)}^{2}-0.8614\cdot \mathrm{Fe}\left(\mathrm{III}\right)\cdot H_{2}O_{2}-10.5792\cdot H_{2}O_{2}^{2} \end{array}$$

(2) $$\begin{array}{l} Removal\;efficiency\;OG\;\left(\%\right)\\ \;=92.9426-2.20419\cdot \mathrm{Polyphenols}+0.95027\cdot \mathrm{Fe}\left(\mathrm{III}\right)+3.81278\cdot H_{2}O_{2}-1.36892\\ \;\cdot \;{\mathrm{Polyphenols}}^{2}-3.92476\cdot \mathrm{Polyphenols}\cdot \mathrm{Fe}\left(\mathrm{III}\right)+2.30976\cdot \mathrm{Polyphenols}\cdot H_{2}O_{2}-1.82448\\ \;\cdot \mathrm{Fe}{\left(\mathrm{III}\right)}^{2}-0.690238\cdot \mathrm{Fe}\left(\mathrm{III}\right)\cdot H_{2}O_{2}-3.59872\cdot H_{2}O_{2}^{2} \end{array}$$

(3) $$\begin{array}{l} Removal\;efficiency\;MO\;\left(\%\right)\\ \;=80.0673-3.56445\cdot \mathrm{Polyphenols}+23.96480\cdot \mathrm{Fe}\left(\mathrm{III}\right)+18.3342\cdot H_{2}O_{2}-2.08636\\ \;\cdot \;{\mathrm{Polyphenols}}^{2}-3.71663\cdot \mathrm{Polyphenols}\cdot \mathrm{Fe}\left(\mathrm{III}\right)+2.51242\cdot \mathrm{Polyphenols}\cdot H_{2}O_{2}-9.5837\\ \;\cdot \mathrm{Fe}{\left(\mathrm{III}\right)}^{2}-17.0007\cdot \mathrm{Fe}\left(\mathrm{III}\right)\cdot H_{2}O_{2}-9.74664\cdot H_{2}O_{2}^{2} \end{array}$$

Analysis of the coded quadratic models shows consistent positive linear effects of H_2_O_2_ across dyes, strongest for methyl orange and methylene blue. Fe(III) has a strong positive effect on methyl orange, moderate on methylene blue, and minimal on orange G. Polyphenols display negative linear coefficients in all models, indicating that lower levels are favorable when other reagents are sufficient. Negative quadratic terms for Fe(III) and H_2_O_2_ suggest diminishing returns and internal optima; orange G also shows negative curvature for polyphenols, while methylene blue shows positive curvature, favoring low polyphenol levels. Interaction effects vary: AB is antagonistic for orange G and methylene blue, AC is synergistic for orange G, and BC is strongly antagonistic for methyl orange and modestly so for methylene blue, implying that high Fe(III) and H_2_O_2_ together can reduce performance.

Finally, Table 4 reports the coded and real values of the operating variables corresponding to the theoretical maximum removal efficiency for each dye, which is predicted to be 100%.

The predicted optima fall within or near the experimental domain and reflect the interaction and curvature patterns. For methylene blue, complete removal is achieved at low polyphenols (−1.35), moderate Fe(III) (+0.62), and high H_2_O_2_ (+0.96), consistent with the strong positive H_2_O_2_ effect and its negative quadratic term. For orange G, the optimum is very low polyphenols (−1.65), very high Fe(III) (+1.59), and near-central H_2_O_2_ (+0.04), aligning with the AB and AC interactions and weaker Fe(III) effect. For methyl orange, complete removal occurs at low polyphenols (−1.29), high Fe(III) (+1.09), and moderately elevated H_2_O_2_ (+0.21), balancing the strong Fe(III)/H_2_O_2_ effects against the negative BC interaction and quadratic terms. Because Fe(III) and H_2_O_2_ show concavity, operation slightly inside the high-factor edges is advisable for robustness. Under the conditions in Table 4, the theoretical optima were confirmed experimentally, yielding complete dye removal.

#### 3.3.2. Graphical Analysis

To complement the numerical analysis, a graphical analysis was conducted to visualize effect magnitudes, response trends, factor interactions, and model adequacy. Four plot types were examined: Pareto charts of standardized effects, main-effects plots, interaction plots, and observed-versus-predicted plots.

**Figure 7:**
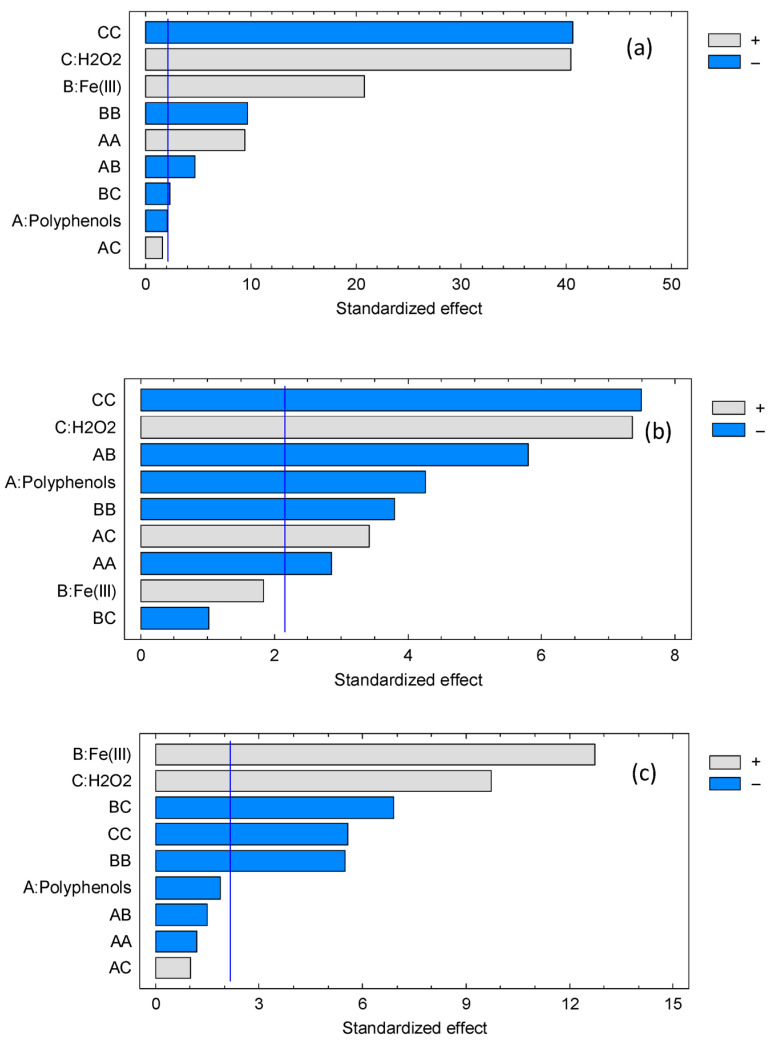
Pareto plots of methylene blue (**a**), orange G (**b**) and methyl orange (**c**).

Figure 7 depicts the Pareto plots corresponding to each of the dyes studied. Pareto charts of standardized effects are used to rank the relative importance of factors and interactions in a design, providing an immediate visual summary that complements the ANOVA table. Bars that exceed the critical significance line identify terms with statistically meaningful contributions, while sub-threshold bars suggest candidates for model simplification.

The Pareto charts confirm the hierarchy of standardized effects across dyes, consistent with ANOVA and the quadratic models. For methylene blue, H_2_O_2_ and Fe(III) dominate, polyphenols fall below significance, and AB and BC interactions are relevant, with strong curvature from the squared terms. This aligns with ANOVA *p*-values (B, C: *p* < 0.001; A: *p* = 0.0576; AB: *p* = 0.0004; BC: *p* = 0.0353; AC: *p* = 0.1295; AA, BB, CC: *p* ≤ 0.001) and the negative quadratic coefficients indicating diminishing returns at high doses.

For orange G, polyphenols and H_2_O_2_ are the strongest main effects, Fe(III) is weaker, and AB and AC are significant; BC remains below the cutoff. The pattern mirrors ANOVA (A: *p* = 0.0009; C: *p* < 0.001; B: *p* = 0.0894; AB: *p* = 0.0001; AC: *p* = 0.0046; BC: *p* = 0.3261; AA, BB, CC: *p* ≤ 0.0136) and reflects the dual role of polyphenols as ligands and reductants that modulate Fe redox cycling.

For methyl orange, Fe(III) and H_2_O_2_ dominate, with a strongly significant BC interaction and large Fe(III) and H_2_O_2_ quadratic terms; polyphenols and other interactions remain nonsignificant. This matches ANOVA (B, C: *p* < 0.001; A: *p* = 0.0809; BC: *p* < 0.001; BB, CC: *p* ≤ 0.0001; AA, AB, AC: *p* ≥ 0.1549) and the antagonistic BC coefficient.

Overall, the Pareto plots reinforce a mechanistic picture in which dye removal is governed mainly by oxidant and catalytic iron, with curvature effects from self-quenching at high levels, and modulation by polyphenols through complexation and redox mediation.

**Figure 8:**
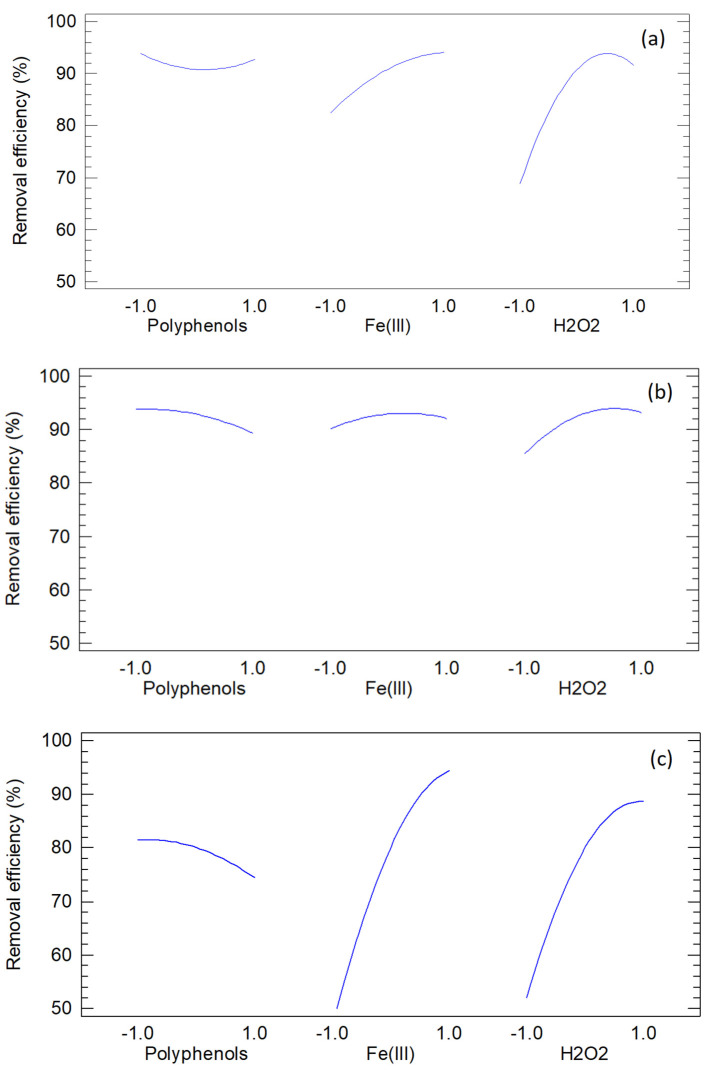
Main effects plots of methylene blue (**a**), orange G (**b**) and methyl orange (**c**).

Figure 8 presents the main-effects plots for the three dyes, showing the marginal influence of each factor averaged over the others. Line direction and steepness indicate whether a factor improves or impairs performance, while curvature reflects quadratic behavior in FCCORD models. These plots highlight sensitive ranges and practical adjustments but should be interpreted alongside ANOVA and Pareto results, since significance is not conveyed by slope alone and interactions can mask or exaggerate marginal effects.

For methylene blue, performance depends strongly on H_2_O_2_, with the steepest gains at low-to-mid levels and attenuation at the highest, consistent with diminishing returns. Fe(III) shows a positive but moderate effect, while polyphenols display a slight negative trend. These patterns align with Fenton-type mechanisms where radical availability is driven mainly by H_2_O_2_, supported by Fe(III), and reduced by polyphenols through radical scavenging or Fe complexation. The curvature in the H_2_O_2_ trace matches the negative quadratic terms identified numerically.

For orange G, H_2_O_2_ shows a strong positive effect on removal, while polyphenols exert a negative influence greater than Fe(III), whose impact appears weak, consistent with its limited statistical significance. Slight flattening at higher factor levels suggests nonproductive oxidant consumption or self-quenching, in line with the quadratic behavior seen in regression. Thus, oxidant availability dominates, while excess polyphenols depress removal via radical scavenging and iron complexation.

For methyl orange, both H_2_O_2_ and Fe(III) display strong positive effects, with the greatest improvements from low to mid levels and diminishing benefit at the highest settings. Polyphenols are neutral to slightly negative, showing no enhancement once sufficient oxidant and catalyst are present. The slope attenuation at high factor levels reflects side reactions from excess oxidant/catalyst that reduce decolorization efficiency. Overall, the main-effects profiles confirm the dominance of H_2_O_2_ and Fe(III) and suggest operating near, but not at, the upper levels to avoid diminishing returns.

**Figure 9:**
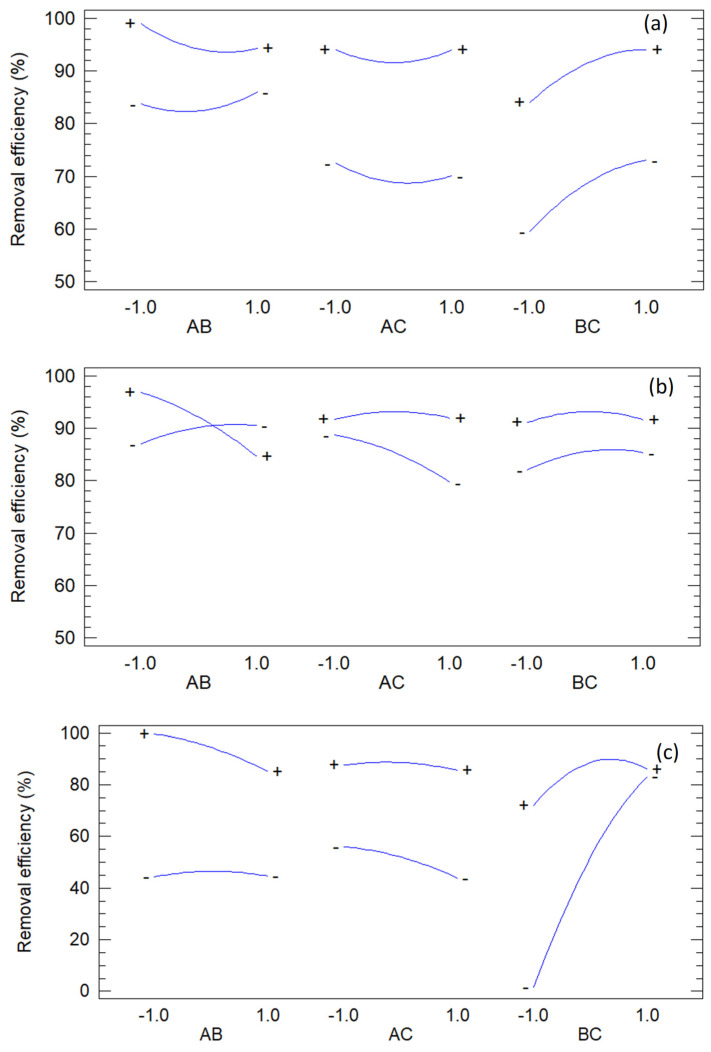
Interaction plots of methylene blue (**a**), orange G (**b**) and methyl orange (**c**).

Figure 9 shows the interaction plots, which diagnose non-additive behavior in FCCORD designs by displaying a factor’s effect at different levels of another. Divergence from parallelism indicates synergistic or antagonistic interactions, corroborating ANOVA and clarifying their practical impact. Crossing or widely separated lines highlight operating regions sensitive to joint factor changes.

For methylene blue, departures from parallelism appear in AB and BC, while AC remains nearly parallel. In AB, the Fe(III) effect depends strongly on polyphenols, with divergent slopes confirming the significant AB term. The BC panel also shows divergence, consistent with competing radical generation and scavenging at high dosages. AC shows no interaction. Collectively, MB removal is favored at moderate Fe(III), high H_2_O_2_, and low polyphenols.

For orange G, AB and AC exhibit clear interactions, while BC remains parallel. In AB, Fe(III)’s effect varies with polyphenols, reflecting Fe–phenolic complexation that enhances redox cycling at low levels but suppresses activity at high concentrations. In AC, divergence shows that polyphenols modulate the benefit of H_2_O_2_, likely through mediation of Fe redox cycling. BC remains additive. These interactions explain why optimal OG removal required very low polyphenols, very high Fe(III), and near-central H_2_O_2_.

For methyl orange, BC dominates, with strong divergence/crossing indicating high sensitivity to Fe(III)–H_2_O_2_ dosing, consistent with its significant BC term. Removal improves when iron and oxidants are balanced, while excess promotes non-productive consumption and antagonism. AB and AC are nearly parallel, showing limited polyphenol influence. This supports the optimum of high Fe(III), moderately elevated H_2_O_2_, and low polyphenols, avoiding interaction-driven efficiency losses at the extreme settings.

Response surface and contour plots visualize the joint effects of two factors while holding the third at its central level, translating the quadratic FCCORD models into an interpretable landscape. Parallel contours indicate additivity, tilted ellipses indicate interaction, and closed vs. open contours distinguish interior from edge optima. Gradient steepness shows sensitivity, while surface flattening reflects diminishing returns. Together with ANOVA and other plots, these surfaces guide the selection of robust operating conditions near the predicted optima.

**Figure 10:**
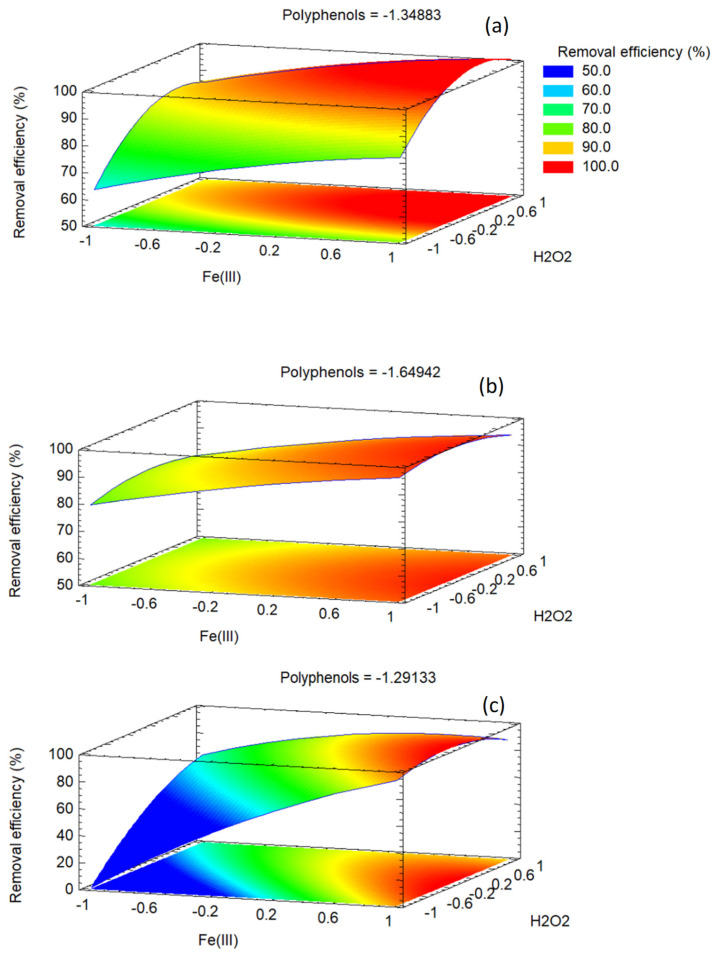
Response surface plots of methylene blue (**a**), orange G (**b**) and methyl orange (**c**). The concentration of polyphenols is always kept at its optimal coded value.

Figure 10 shows the response surface plots for the three dyes under study. In all cases, the concentration of polyphenols was kept at its optimal value. With polyphenols fixed at the optimal coded level for each dye, the B–C response surfaces (B = Fe(III), C = H_2_O_2_) visualize how oxidant and catalyst jointly control performance at the most favorable polyphenol setting.

For methylene blue, the surface shows a dome-shaped region with the maximum near moderate Fe(III) and relatively high H_2_O_2_, consistent with the significant B×C interaction and negative quadratic terms. Contours are closer along the H_2_O_2_ axis, indicating greater sensitivity to oxidant. At simultaneous high Fe and H_2_O_2_, removal declines due to non-productive radical consumption. The optimum lies within the domain, with a compact elliptical zone indicating robustness.

For orange G, fixing polyphenols at low levels yields a largely additive surface, consistent with the non-significant B × C term. The gradient is dominated by Fe(III), with responses forming a plateau at high iron, while H_2_O_2_ has a modest influence near its center. Curvature along the Fe(III) axis reflects the strong squared term, while H_2_O_2_ shows mild curvature. The maximum occurs at very high Fe(III) and near-central H_2_O_2_, reinforcing the iron-driven behavior once polyphenols are minimized.

For methyl orange, the surface is shaped by the B × C interaction, producing a bent ridge where high Fe(III) must pair with moderate H_2_O_2_ for maximum removal. Performance declines at simultaneous high levels, consistent with antagonism from radical self-quenching. Steep contours near the optimum indicate sensitivity to H_2_O_2_ when Fe is high. Negative curvature in both B and C confines the high-response region, placing the optimum near high Fe(III) and moderate H_2_O_2_ rather than at the design corners.

### 3.4. Some Insights into the Degradation Mechanism of Dyes

The nZVI synthesized from coffee waste offers a promising approach for environmental remediation by degrading dyes in aqueous systems. However, the complexity of these systems, due to the presence of organic compounds such as coffee polyphenols, generates multiple reactive species that interact with the nanoparticles and hydrogen peroxide (H_2_O_2_), creating a network of reactions that is difficult to trace. This issue is further complicated by the rapid reaction rates, which not only make it challenging to quench the reaction at specific time intervals but also to capture and analyze intermediate products accurately using conventional techniques such as HPLC-MS.

Polyphenols derived from waste coffee play a dual role in both the synthesis and stabilization of zero-valent iron nanoparticles, as well as in the degradation of dyes. During synthesis, polyphenols act as reducing agents, facilitating the conversion of Fe(III) to Fe(0), which leads to the formation of the nanoparticles (Equation (4)). At the same time, due to their antioxidant properties, they prevent excessive oxidation and aggregation, thereby stabilizing the nZVI.

Fe^3+^ + Polyphenol → Fe(0) + Oxidized polyphenol (4)

Simultaneously, polyphenols facilitate the transformation (Equation (5)) of Fe(III) into Fe(II), a species necessary for the production of hydroxyl radicals involved in the Fenton-like reactions catalyzed by nZVI when hydrogen peroxide is introduced into the reaction medium (Equation (6)). In parallel, the iron oxide or oxohydroxide layer formed on the nZVI surface due to partial oxidation contributes to the catalytic activity by facilitating the disproportionation of hydrogen peroxide into hydroxyl radicals and hydroxide ions (Equation (7)). The hydroxyl radicals generated are highly reactive and essential for the degradation of dye pollutants in water (Equation (8)).

Fe^3+^ + Polyphenol → Fe^2+^ + Oxidized polyphenol (5)

Fe^2+^ + H_2_O_2_ → Fe^3+^ + ▪ OH + OH^−^ (6)

2 FeOOH + H_2_O_2_ → Fe_2_O_3_ + ▪ OH + OH^−^ + H_2_O (7)

▪ OH + Dye → Degraded products (8)

The nZVI demonstrates a moderate surface area, which enhances its effectiveness as an adsorbent for dye molecules. This extensive surface area allows the nanoparticles to capture a significant amount of dye from aqueous solutions, directly reducing the dye concentration through adsorption. In some cases, adsorption may serve as a key removal mechanism, while in others, it could act as a preliminary step that concentrates dye molecules on the nanoparticle surface, bringing them closer to the reactive sites and potentially increasing their susceptibility to degradation by hydroxyl radicals.

The results from desorption experiments indicate that methylene blue is partially removed by adsorption, as it was later detected back in the solution. In contrast, the failure to observe desorption for methyl orange and orange G suggests that, although these azo dyes may adsorb onto the nanoparticle surface, they degrade more efficiently and rapidly. This is likely due to the high reactivity of the azo bond (–N=N–) with hydroxyl radicals, which facilitates its cleavage and initiates a cascade of oxidative reactions. As a result, the dyes are quickly broken down, leaving little opportunity for intact molecules to desorb back into the solution.

There are other reasons that can justify this difference in behavior. The nZVI are typically considered to carry a negative charge in aqueous solution, unless the pH is sufficiently acidic [45]. This occurs because they react quickly with water and dissolved oxygen, forming a surface layer of iron oxides or hydroxides [46] (Equation (9)), which results in a net negative surface charge. This strongly promotes the adsorption of positively charged particles such as methylene blue cations through electrostatic attractive interactions (Equation (10)), whereas negatively charged dyes like methyl orange and orange G do not experience this type of interaction.

Fe^3+^ + 3 OH^−^ → Fe(OH)_3_ → FeOOH + H_2_O (9)

FeOOH + MB cations (or intermediates) → FeOOH–MB(10)

**Figure 11:**
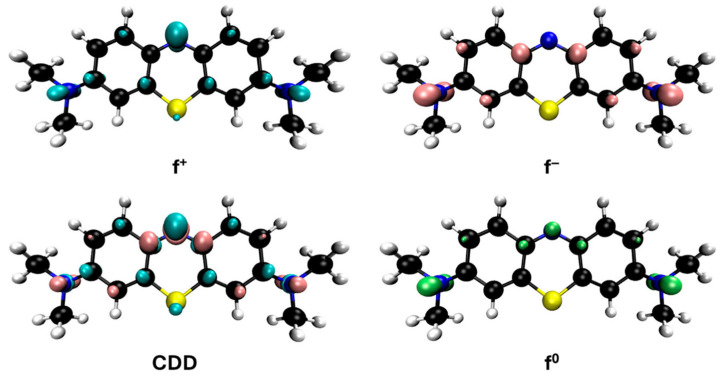
Reactivity map of methylene blue cation based on Fukui functions, showing isosurfaces at 0.005 a.u. that highlight areas susceptible to acting as an electrophile (blue), nucleophile (pink), and in free radical reactions (green). Atom color coding: C (black), H (white), N (blue), S (yellow).

However, since adsorption is much slower than radical oxidation and given the rapid disappearance of the solution’s color upon the addition of hydrogen peroxide, it can be considered that methylene blue is primarily degraded by hydroxyl radicals. Some studies on the degradation of this dye using Fenton-like and photo-Fenton methods [47,48,49,50,51,52] have proposed intermediates based on the mass spectra obtained from HPLC-MS analysis. These proposals, although varied, often involve consecutive demethylations of the nitrogen atoms in the methylene blue molecule through compounds such as azure A, azure B, azure C and thionine, as well as hydroxylated derivatives of these species. This mechanism can be supported from the perspective of DFT calculations by Fukui functions, which allow for a graphical analysis of the cation reactivity corresponding to these salts in solution (Figure 11). These functions are represented by isosurfaces that indicate the regions of the molecule most prone to act as electrophilic (f^+^), nucleophilic (f^−^), or, in this particular case, as if they are undergoing radical reactions, described by the f^0^ function. The condensed dual descriptor (CDD) combines the nucleophilic and electrophilic character of different molecular regions into a single representation. As shown in Figure 11, the amino groups attached to the methyl groups in methylene blue highlight the most reactive areas for free radical reactions (f^0^), supporting that an important degradation mechanism proceeds through demethylation. The f^0^ functions of the derivatives mentioned above are also shown in Figure S1 and are quite similar.

Simultaneously, this set of cations may undergo cleavage of the central ring by radical attack, leading to simpler aromatic compounds that would eventually oxidize further to complete mineralization into CO_2_, H_2_O and inorganic ions, through less colored and less toxic organic molecules. References [48,49] suggest that the cleavage of the central ring leads to the detection of characteristic signals during degradation, which are associated with benzothiazole, catechol and 4-aminocatechol. While we have highlighted that identifying compounds within the complexity of our reaction medium is highly challenging, we have conducted a proof-of-concept experiment by generating nZVI under equivalent conditions with NaBH_4_ and analyzing the HPLC-MS spectrum after the coloration disappeared (Figure S2). Obviously, our case involves different conditions compared to the ones described in the referenced articles, starting with the timing of the quenching to stop the reaction. Nevertheless, we identified [M + H]^+^ peaks in the spectrum with intermediate signal intensities, which could be associated with benzothiazole (*m*/*z* 136.02) and catechol (*m*/*z* 111.05).

In the case of azo dyes such as methyl orange and orange G, hydroxyl radicals are expected to attack the azo bond as we said, causing its cleavage and leading to the formation of aromatic amines, with both the dyes and their decomposition products being susceptible to simultaneous hydroxylation and further oxidative degradation. Some articles study this breakdown in these types of processes for methyl orange and orange G [52,53,54,55], which can be summarized according to Equation (11).

▪ OH + R–N = N–R′ → R–NH_2_ + R′–NH_2_ (11)

**Figure 12:**
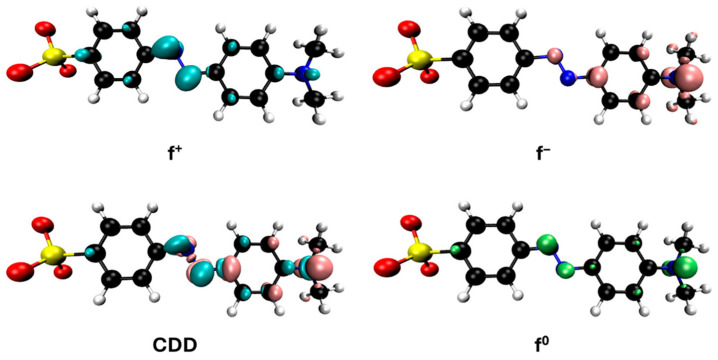
Reactivity map of methyl orange anion based on Fukui functions, showing isosurfaces at 0.005 a.u. that highlight areas susceptible to acting as an electrophile (blue), nucleophile (pink), and in free radical reactions (green). Atom color coding: C (black), H (white), N (blue), O (red), S (yellow).

**Figure 13:**
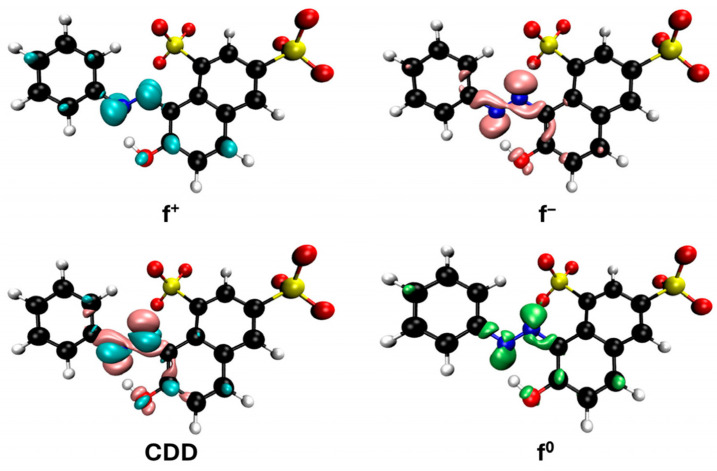
Reactivity map of orange G dianion based on Fukui functions, showing isosurfaces at 0.005 a.u. that highlight areas susceptible to acting as an electrophile (blue), nucleophile (pink), and in free radical reactions (green). Atom color coding: C (black), H (white), N (blue), O (red), S (yellow).

The representations of the Fukui functions f^0^ for these dyes (Figure 12 and Figure 13) support the radical attack on the azo group. Notably, in the case of methyl orange, we can also observe that the attack on the amino group would be quite favorable, leading to simultaneous demethylations as suggested elsewhere [52].

In summary, the nZVI synthesized from coffee waste demonstrates significant potential for dye degradation in aqueous media. Coffee polyphenols play a dual role as reducing and stabilizing agents, enhancing nanoparticle formation and activity. Additionally, DFT calculations using Fukui functions provide valuable insights into the radical-driven molecular mechanisms, supporting the effectiveness of this approach for water treatment.

## 4. Conclusions

From the results obtained in this work, the following conclusions may be drawn:

1. It was established that spent coffee waste provides an effective green precursor for the biosynthesis of nanoscale zero-valent iron, with polyphenols supplying the reducing capacity required for in situ nanoparticle formation and subsequent dye removal.
2. A core–shell architecture was supported by microscopy and elemental mapping, in which a zero-valent iron core is enveloped by an iron-oxide passivation layer and an outer polyphenol-derived carbonaceous coating. This configuration enhances interfacial reactivity while allowing some aggregation.
3. Spectroscopic and diffractometric evidence indicated very poor crystallinity and the presence of oxygenated and phenolic surface functionalities. Broad diffuse features and attenuated iron reflections were consistent with extremely small iron domains below routine laboratory detection limits.
4. The face-centered central composite design at an alpha level of 0.05 identified hydrogen peroxide and Fe(III) as the principal drivers of removal for methylene blue and methyl orange, whereas hydrogen peroxide and polyphenols were most influential for orange G, with Fe(III) playing a lesser role.
5. Statistically significant interactions were dye-dependent: AB and AC interactions were confirmed for orange G, and a strong BC interaction was confirmed for methyl orange. Negative quadratic terms for Fe(III) and hydrogen peroxide indicated diminishing returns at high levels, supporting interior or near-edge optima rather than corner solutions.
6. Operating conditions that achieved complete removal were determined and experimentally verified. Robust windows were identified near, but not at, the highest levels of oxidant and iron, which limited nonproductive radical consumption and preserved performance against small perturbations.
7. The mechanistic approach is coherent with a radical-mediated degradation pathway under Fenton and Fenton-like conditions. Hydroxyl radical attack accounted for demethylation and subsequent ring opening in methylene blue, and for azo-bond cleavage in methyl orange and orange G. Polyphenols acted as reductants during synthesis and as ligands that modulate Fe(III) to Fe(II) cycling during treatment; at elevated concentrations, they also behaved as radical scavengers, explaining the negative main effect observed in the statistical analysis.
8. Practical implications include the feasibility of valorizing coffee waste into reactive iron materials for water decolorization, together with the need to manage aggregation and to validate performance in realistic water matrices, where ionic strength, natural organic matter, and competing oxidant demand may influence efficiency and iron release.
9. The development of efficient methods for processing and utilizing coffee waste and by-products constitutes an important effort to minimize food waste and reuse it in eco-friendly processes, centered on a circular economy approach.

## Figures and Tables

**Table 1:** Experimental design matrix and removal efficiencies of the three dyes.

Concentration (Coded Value)			Removal Efficiency (%)		
Polyphenols	Fe(III)	H_2_O_2_	MB	OG	MO
−1	−1	1	98.09	83.09	68.3
0	0	0	90.76	93.26	79.6
−1.68179	0	0	90.50	95.11	74.8
0	0	0	90.60	93.44	83.0
0	0	0	97.82	93.44	80.7
0	0	0	91.02	89.00	81.4
−1	1	1	93.29	92.89	85.6
0	0	0	95.74	93.07	77.6
0	1.68179	0	72.31	89.74	95.9
1.68179	0	0	90.71	83.81	66.9
0	−1.68179	0	95.03	86.60	3.4
0	0	0	90.97	93.44	78.1
1	1	1	64.28	85.48	73.4
0	0	0	88.6	93.63	78.9
1	−1	−1	91.80	80.71	4.8
1	−1	1	73.16	94.19	72.9
0	0	0	62.51	93.44	82.9
1	1	−1	90.76	77.57	75.2
−1	−1	−1	80.06	81.66	12.2
0	0	0	79.32	93.63	79.5
0	0	1.68179	39.98	91.41	90.2
−1	1	−1	98.09	92.31	95.6
0	0	−1.68179	90.76	74.90	8.2

**Table 2:** Identification and assignment of the peaks in the chromatogram of the coffee waste extract.

Peak	Retention Time (min)	Compound	Structure
1	2.06	Gallic acid	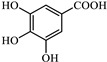
2	2.39	Protocatechuic acid	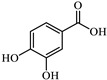
3	3.50	Vanillic acid	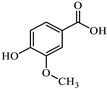
4	7.78	Caffeic acid	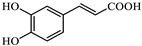
5	11.15	Caffeine	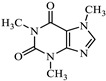
6	11.75	Ferulic acid	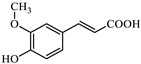
7	12.95	p-Coumaric acid	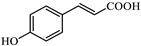
8	14.64	Chlorogenic acid (isomer 1)	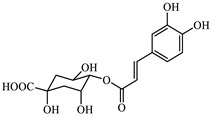
9	17.54	Chlorogenic acid (isomer 2)	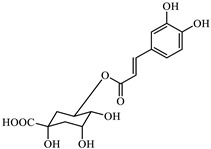

**Table 3:** Results of the ANOVA test for the three dyes under study.

Factor	Methylene Blue	Orange-G	Methyl Orange
A: Polyphenols	0.0576	0.0009	0.0809
B: Fe(III)	0.0000	0.0894	0.0000
C: H_2_O_2_	0.0000	0.0000	0.0000
AA	0.0000	0.0136	0.2535
AB	0.0004	0.0001	0.1549
AC	0.1295	0.0046	0.3259
BB	0.0000	0.0022	0.0001
BC	0.0353	0.3261	0.0000
CC	0.0000	0.0000	0.0001

**Table 4:** Coded and real values of the operating variables leading to the total removal of the dyes.

Variable	Coded Values			Real Values		
	MB	OG	MO	MB	OG	MO
Polyphenols	−1.34883	−1.64942	−1.29133	55.44	5.85	64.93
Fe(III)	0.618576	1.58924	1.08589	2.74 × 10^−2^	3.91 × 10^−2^	3.30 × 10^−2^
H_2_O_2_	0.963645	0.0361344	0.208298	1.58 × 10^−2^	1.02 × 10^−2^	1.12 × 10^−2^

## Data Availability

The original contributions presented in this study are included in the article/Supplementary Material. Further inquiries can be directed to the corresponding authors.

## Supplementary Materials

The following supporting information can be downloaded at: https://www.mdpi.com/article/10.3390/jox15050158/s1, Section S1. Materials and Methods. Figure S1. Reactivity map of ions of azure A, azure B, azure C, and thionine based on f0Fukui functions, showing isosurfaces at 0.005 a.u., which highlight areas susceptible to free radical reactions (green); Figure S2. HPLC-MS analysis using ethyl acetate as the solvent for methylene blue degraded via a Fenton-like process with nZVI synthesized using NaBH_4_, after color loss. The figure highlights the most representative retention time (tr, in minutes) peaks within the *m*/*z* range of 100–140; Table S1. Coded and real values of the operational variables and experimental design matrix; Table S2. Cartesian coordinates for the optimized structure of methylene blue cation at the M06-2X/6-311++G(3df,3pd) level with SMD solvation model in water; Table S3. Cartesian coordinates for the optimized structure of methyl orange anion at the M06-2X/6-311++G(3df,3pd) level with SMD solvation model in water; Table S4. Cartesian coordinates for the optimized structure of orange G dianion at the M06-2X/6-311++G(3df,3pd) level with SMD solvation model in water; Table S5. Energetic data for optimized structures of studied dyes in their ionic forms (M06-2X/6-311++G(3df,3pd) level with SMD solvation model in water).
