# Supplementary material for: Coffee Waste as a Green Precursor for Iron Nanoparticles: Toward Circular, Efficient and Eco-Friendly Dye Removal from Aqueous Systems
Source: J Xenobiot. 2025 Oct 2;15(5):158. doi: 10.3390/jox15050158 (PMC12565063; doi:10.3390/jox15050158)
Supplement: Supplementary file 1 [file jox-15-00158-s001.zip › jox-3857333-supplementary.pdf]

S1. Materials and methods

S1.1. HPLC analysis

Polyphenols were separated and identified using an HPLC system (Agilent Technologies 1260 Series, Santa Clara, CA, USA) equipped with a UV–Vis photodiode array detector (DAD) and a Luna C18 column (3 µm, 150 × 2.0 mm, 100 Å; Phenomenex, Torrance, CA, USA). Two mobile phases were used. The first one (A) comprised of 0.1% formic acid in water (v/v), while the second one (B) consisted of 0.1% formic acid in methanol. To achieve separation, various gradients were applied throughout the experiment with the following intervals: 0–3 min, 10% B; 3–30 min, 50% B; 30–45 min, 60% B; 45–50 min, 100% B; 50–60 min, 10% B. A constant flow rate of 1 $mL{\cdot}min^{-1}$ was maintained for all analyses. The column temperature was set at a constant 30 °C, and the injection volume was 20 µL. The DAD operated at λ = 280 nm for detection purposes.

S1.2. Folin–Ciocalteu Assay

For the quantification of total polyphenols, the Folin–Ciocalteu assay was employed using UV–Vis spectrophotometry [31]. The Folin–Ciocalteu reagent, containing sodium tungstate and sodium molybdate in a phosphoric acid solution, reacts with phenolic compounds under basic conditions achieved by adding Na2CO3. A yellow phosphomolybdotungstic acid complex forms in acidic medium and, upon reduction by phenolic groups, yields an intense blue complex. The absorbance of this complex was recorded with a Shimadzu UV-1800 UV/Visible scanning spectrophotometer (Cole-Parmer, United States).

Quantification was performed using a gallic acid standard curve, and polyphenol concentrations were expressed as gallic acid equivalents (GAE). Standards (2.3–7.9 mg·L-1) were prepared from a 196 mg·L-1 stock solution in 25 mL volumetric flasks. To each standard, 0.5 mL Folin–Ciocalteu reagent and 10 mL of 7.5% (w/v) Na2CO3 were added, and the volume was brought to 25 mL with ultrapure water. The flasks were kept in the dark for 1 h, after which absorbance was measured at 760 nm against a reagent blank prepared identically but without gallic acid.

After calibration, polyphenols in the coffee-waste extract were quantified by treating three aliquots of the extract (50, 75, and 100 µL) as above: 0.5 mL Folin–Ciocalteu reagent and 10 mL of 7.5% (w/v) Na2CO3 were added, the volume was adjusted to 25 mL with ultrapure water, the mixtures were incubated in the dark for 1 h, and absorbance was measured at 760 nm.

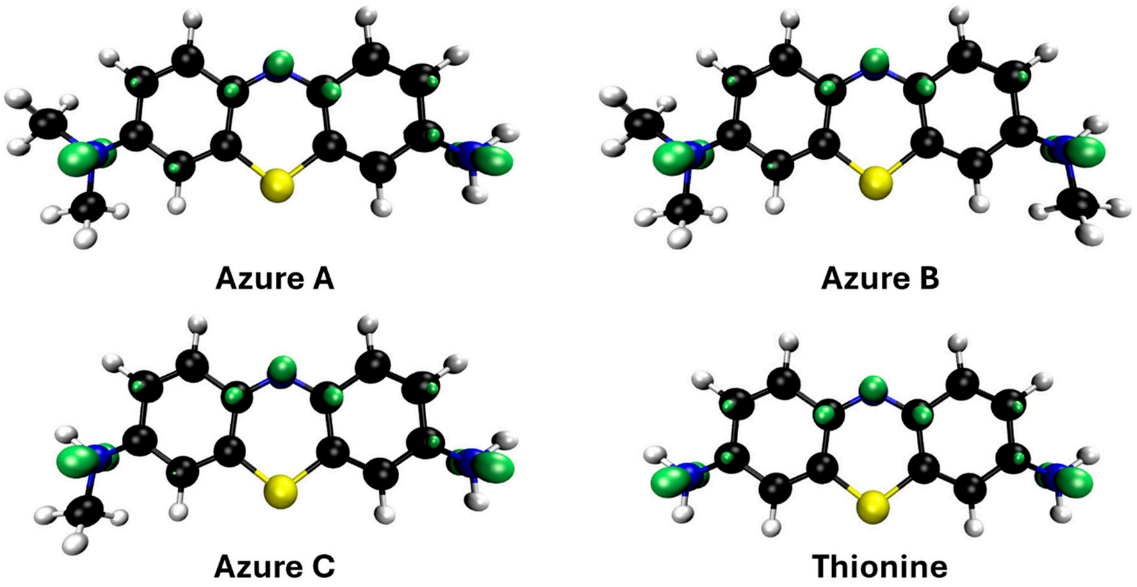

Figure S1. Reactivity map of ions of azure A, azure B, azure C, and thionine based on f^0Fukui functions, showing isosurfaces at 0.005 a.u., which highlight areas susceptible to free radical reactions (green).

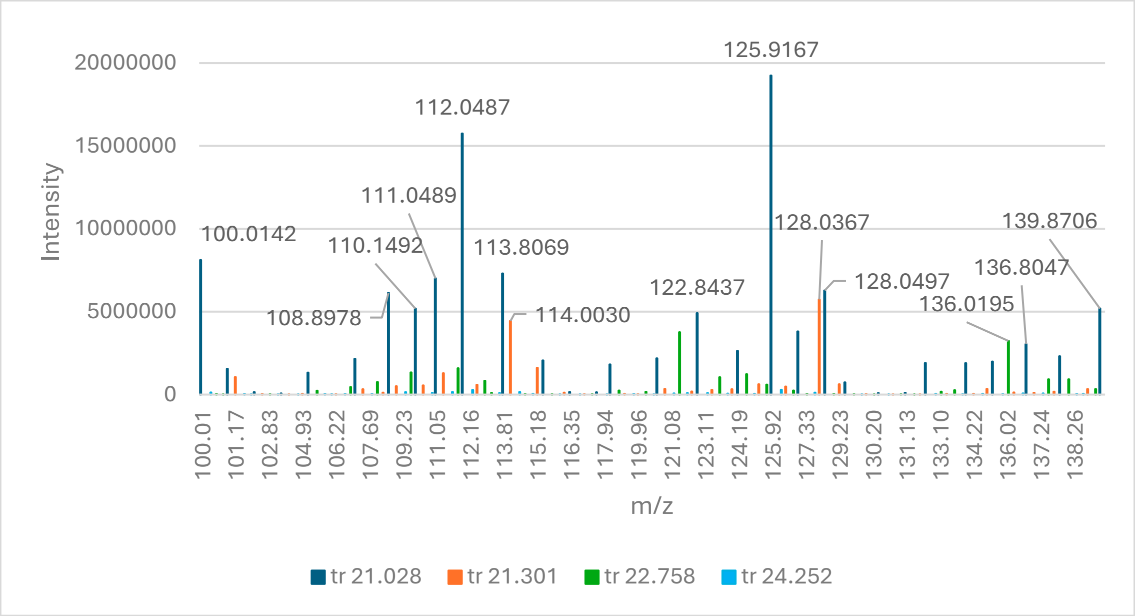

Figure S2. *HPLC-MS analysis using ethyl acetate as the solvent for methylene blue degraded via a Fenton-like process with nZVI synthesized using NaBH4, after color loss. The figure highlights the most representative retention time (tr, in minutes) peaks within the m/z range of 100-140.*

Table S1. Coded and real values of the operational variables and experimental design matrix.

**Variable**	**Coded value**				
	**-1.68179**	**-1**	**0**	**1**	**1.68179**
Polyphenols (mg·L^{-1})	0	113	278	443	556
Fe (III) (M)	0	$8.1\cdot10^{-3}$	$2.0\cdot10^{-2}$	$3.2\cdot10^{-2}$	$4.0\cdot10^{-2}$
H_2O_2 (M)	0	$4.1\cdot10^{-3}$	$1.0\cdot10^{-2}$	$1.6\cdot10^{-2}$	$2.0\cdot10^{-2}$

Table S2. Cartesian coordinates for the optimized structure of methylene blue cation at the M06-2X/6-311++G(3df,3pd) level with SMD solvation model in water.

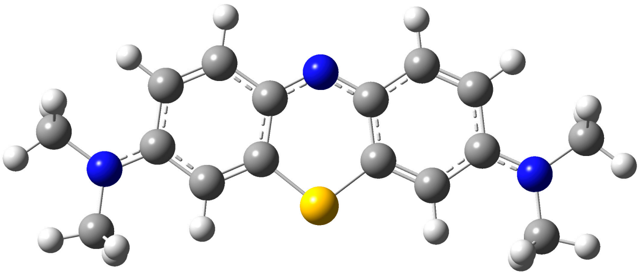

1 1

ATOM TYPE	x	y	z
C	-3.59014900	1.41868600	-0.00000700
C	-3.76836900	-0.00717900	-0.00003100
C	-2.61375600	-0.82757700	-0.00003000
C	-1.35932200	-0.26767600	-0.00003200
C	-1.17143700	1.15181000	-0.00002600
C	-2.34931100	1.95625000	-0.00000900
S	-0.00000100	-1.33419800	-0.00004100
C	1.35932200	-0.26768500	-0.00003700
C	1.17143400	1.15181200	-0.00003300
N	0.00000700	1.77513600	-0.00002600
C	2.61375300	-0.82758200	-0.00003300
C	3.76837000	-0.00718200	-0.00004000
C	3.59015200	1.41868800	-0.00001700
C	2.34932000	1.95625200	-0.00002000
N	4.99221900	-0.53793100	-0.00005500
N	-4.99222600	-0.53793100	-0.00004700
C	-5.16783200	-1.98533200	0.00010900
C	-6.17703500	0.31495400	0.00004900
C	6.17703400	0.31495300	0.00015200
C	5.16782900	-1.98533600	0.00003500
H	-4.45160100	2.06658700	0.00002400
H	-2.71400500	-1.90201400	-0.00003600
H	-2.21296000	3.02868900	0.00001100
H	2.71400700	-1.90201900	-0.00001500
H	4.45160800	2.06658500	0.00000100

H	2.21296500	3.02869000	-0.00000800
H	-4.71485000	-2.43015900	-0.88558000
H	-4.71490900	-2.42996700	0.88592700
H	-6.22754400	-2.20847400	0.00009400
H	-6.20149100	0.94741600	0.88649900
H	-6.20139300	0.94773600	-0.88617000
H	-7.05623200	-0.31725400	-0.00012700
H	7.05622900	-0.31725600	0.00011400
H	6.20149900	0.94769900	-0.88609100
H	6.20137800	0.94744800	0.88658000
H	4.71470700	-2.43013000	-0.88559500
H	6.22754100	-2.20847500	-0.00016400
H	4.71504500	-2.42999700	0.88591200

Table S3. Cartesian coordinates for the optimized structure of methyl orange anion at the M06-2X/6-311++G(3df,3pd) level with SMD solvation model in water.

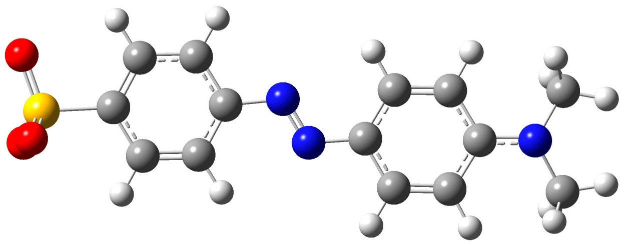

ATOM TYPE	x	y	z
-1 1			
C	5.17931200	-0.05014300	0.08614900
C	4.52542000	-1.30050000	0.05773300
C	3.14862000	-1.37184800	0.04278100
C	2.36158300	-0.22117900	0.04494100
C	2.99725500	1.02600400	0.05341600
C	4.36905100	1.11308700	0.06808800
N	6.54189100	0.03871400	0.13762100
C	7.17361600	1.31734300	-0.15169100
C	7.32703800	-1.15541700	-0.13351300
N	0.97231500	-0.42135700	0.02802100
N	0.26306000	0.60065000	0.03842400
C	-1.13786100	0.35522600	0.02206300
C	-1.94760000	1.48383100	0.01845600
C	-3.33085000	1.35598600	0.00332300
C	-3.89537200	0.09249300	-0.00724800
C	-3.08866500	-1.04518800	-0.00273800
C	-1.71484200	-0.91838100	0.01183000
S	-5.65988400	-0.12729500	-0.02437800
O	-6.24237500	1.20733100	-0.03338800
O	-5.97000200	-0.88009200	1.18683900

O	-5.94621100	-0.88592000	-1.23783200
H	5.09550700	-2.21537500	0.04729800
H	2.65829700	-2.33628000	0.02366400
H	2.40630500	1.93004500	0.04202600
H	4.82544000	2.09023000	0.06630000
H	6.92639100	1.67716500	-1.15456100
H	6.87311700	2.07343000	0.57050800
H	8.24918900	1.19762800	-0.07908700
H	7.11352900	-1.93476400	0.59519600
H	7.13233500	-1.55372300	-1.13365700
H	8.37980100	-0.90627900	-0.05542500
H	-1.48559400	2.46138300	0.02725600
H	-3.96154900	2.23259800	0.00015300
H	-3.54042000	-2.02813200	-0.01039100
H	-1.08659400	-1.79568900	0.01582500

Table S4. Cartesian coordinates for the optimized structure of orange G dianion at the M06-2X/6-311++G(3df,3pd) level with SMD solvation model in water.

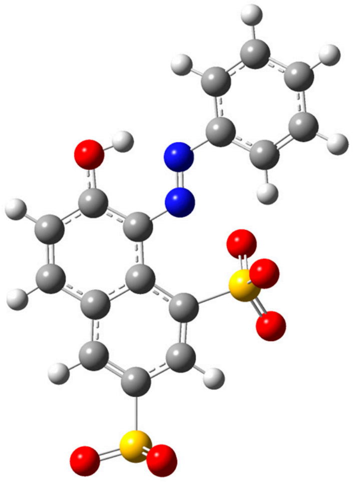

ATOM TYPE	x	y	z
-2 1			
C	0.10818100	3.71252500	0.22180100
C	1.36582800	3.23083200	0.07402800
C	1.62324300	1.83489400	0.01672700
C	0.55315800	0.90256100	0.11398400
C	-0.78260900	1.43309700	0.14272700
C	-0.98112800	2.81536700	0.25610800
C	2.95769300	1.40839400	-0.14088200

C	3.24904900	0.07890600	-0.16995300
C	2.22699200	-0.85914000	0.04145700
C	0.91734800	-0.48089900	0.20399800
O	-2.19667100	3.35930100	0.33932700
N	-1.83922700	0.55283600	-0.09968300
N	-2.99185700	0.99741000	0.08374500
C	-4.06889700	0.12444400	-0.20615500
C	-3.90714800	-1.23280000	-0.47933200
C	-5.02852500	-2.00471100	-0.73413600
C	-6.29927400	-1.43514000	-0.71954800
C	-6.45171900	-0.08317900	-0.44005500
C	-5.33630700	0.69785100	-0.17651600
S	4.90816700	-0.51094200	-0.39727200
O	4.86174900	-1.34655200	-1.59253100
O	5.22071800	-1.27685300	0.80425800
O	5.74085300	0.67304400	-0.55781000
S	-0.17233900	-1.84922500	0.65278900
O	-1.09113900	-1.34557400	1.66386500
O	-0.81307100	-2.30019200	-0.57607800
O	0.72214400	-2.86550300	1.19630900
H	-0.10142700	4.77072000	0.28426700
H	2.20809300	3.90614000	0.00632900
H	3.73570700	2.15293100	-0.23728500
H	2.48494700	-1.90489200	0.11877500
H	-2.84986100	2.60147500	0.32533400
H	-2.91557900	-1.66182300	-0.47943600
H	-4.91407200	-3.05945800	-0.94309400
H	-7.16745400	-2.04700400	-0.92122700
H	-7.43665500	0.36168400	-0.42362400
H	-5.43133400	1.75178300	0.04900500

Table S5. Energetic data for optimized structures of studied dyes in their ionic forms (M06-2X/6-311++G(3df,3pd) level with SMD solvation model in water).

Molecule	Electronic energy (kJ/mol)	Free energy (kJ/mol)	Entropy (J/(mol·K))	Enthalpy (kJ/mol)	EHOMO (eV)	ELUMO (eV)	ΔEH-L (eV)
Methylene blue	-3105310,92	-3104603,08	578,67	-3104430,55	-6,78	-2,72	4,06
Methyl orange	-3492227,95	-3491655,86	623,92	-3491469,84	-6,66	-1,55	5,10
Orange G	-5378243,56	-5377725,60	665,80	-5377527,09	-7,22	-1,73	5,49
